# Prevalence of hyperinsulinemia and its association with measures of adiposity and body composition in 16-25-year-old adolescents and young adults in Mumbai

**DOI:** 10.1186/s12902-026-02230-0

**Published:** 2026-03-13

**Authors:** Panchali Moitra, Jagmeet Madan, Soumik Kalita, Sharvari Desai, Shobha A. Udipi, Rama Vaidya

**Affiliations:** 1 Department of Postgraduate Programs and Research, Sir Vithaldas Thackersey College of Home Science (Empowered Autonomous Status), Shreemathi Nathibai Damodar Thackersey Women’s University, Santacruz West, Mumbai, Maharashtra 400049 India; 2 FamPhy, Gurugram, Haryana India; 3 Kasturba Health Society-Medical Research Center, Mumbai, India

**Keywords:** Adolescents and young adults, Central adiposity, Diabetes in youth, Hyperinsulinemia, India, Insulin resistance, Obesity in adolescents, Prediabetes, Prevalence of insulin resistance

## Abstract

**Background:**

Hyperinsulinemia and insulin resistance (IR) are early indicators of metabolic dysfunction that often precede the development of Type 2 Diabetes (T2D). Identifying these conditions in apparently healthy youth—who may not yet exhibit clinical symptoms—is essential for early intervention. Therefore, we conducted this community-based study to determine the prevalence of hyperinsulinemia, IR, and their associations with measures of adiposity and body composition in 16-25-year-old adolescents and young adults in Mumbai, India.

**Methods:**

Participants (*n* = 1313, M_age_ 19.6 (2.1) years, 65.2% females) reported socio-demography, medical history, and physical activity levels. Hyperinsulinemia was classified as fasting insulin ≥ 15mIU/ml and/or stimulated insulin ≥ 80 mIU/ml and IR as HOMA-IR > 2.5. Anthropometry and blood pressure were measured. Sex-adjusted logistic regression analyses were performed to examine the association of adiposity measures with hyperglycemia and hyperinsulinemia. The odds ratios (ORs) and their 95% confidence intervals (CI) were calculated, significant at *p* ≤ 0.05.

**Results:**

The prevalence of hyperinsulinemia, IR, overweight, and obesity was 39.7%, 17.3%, 15.0%, and 20.5% respectively. Of those with prediabetes (4.5%), more than half (58.1%) were overweight and almost one-third (29.1%) had abdominal obesity. The prevalence of abdominal obesity was 15.1% in participants with normal weight and 36.4% in those with overweight or obesity. Additionally, we observed that 30.1% of participants who were normal or underweight had hyperinsulinemia, and one-third of those with IR were neither overweight nor obese. The odds of hyperglycemia increased from 2.41 times in individuals with overweight (95% CI 1.91, 6.71, *p* = 0.03) to 4.67 times in those with obesity (95% CI 1.33, 6.37, *p* = 0.01). Hyperinsulinemia showed significant associations with body fat percent (OR 1.13 (95% CI 1.02, 1.29) *p* = 0.006), waist-to-height ratio (OR 1.66 (95% CI,1.15, 3.30), *p* = 0.02), and blood pressure (OR 1.08, 95% CI 1,03, 1.20, *p* = 0.04).

**Conclusions:**

The high prevalence of hyperinsulinemia and IR in adolescents and young adults and their associations with obesity and elevated blood pressure highlight a clustering of cardiometabolic risk factors. The findings related to the prevalence of hyperinsulinemia and abdominal obesity in participants with normal weight suggest a need for comprehensive screening and further research to develop targeted interventions.

**Clinical trial number:**

Not applicable.

**Supplementary Information:**

The online version contains supplementary material available at 10.1186/s12902-026-02230-0.

## Background

Globally, there has been an increase in the burden of T2D among youth [1, 2], paralleling an increase in the prevalence of overweight and obesity [3]. The clinical manifestations of T2D are often preceded by an asymptomatic, preclinical stage, referred to as insulin resistance (IR) that is characterized by an elevated plasma insulin level or hyperinsulinemia and/ or a greater resistance to the insulin-mediated glucose uptake into the tissue cells [4, 5]. This pathological state can adversely impact multiple target organs and increase the risk of metabolic abnormalities such as impaired lipoprotein profiles, elevated triglycerides, and blood pressure, early in the progression to T2D [6–8]. However, as hyperinsulinemia and IR are not yet considered disorders but precursors for the development of T2D, these conditions are often missed and inadequately addressed in terms of prevention or management, especially among the younger population. Hence, it is imperative that these conditions are identified at an earlier age and the correlates of hyperinsulinemia and IR are better understood.

Evidence from observational and experimental studies suggests a direct association between hyperinsulinemia, IR, and obesity [9–11]. While researchers still debate whether IR contributes to hyperinsulinemia or vice versa [8, 12, 13], there is a consensus that hyperinsulinemia might be a manifestation of a combination of genetic predisposition and poor dietary and lifestyle choices that get precipitated as a compensatory increased secretion of insulin and suppression of hepatic insulin clearance. Hyperinsulinemia triggers an imbalance in the insulin- Growth Hormone- Insulin-like Growth Factor (insulin-GH-IGF) axis, which results in a disruption of the energy metabolism, increasing the risk of higher fat accumulation and obesity [13]. On the other hand, obesity can trigger IR through mechanisms that include but are not limited to a decline in the insulin-stimulated transport of glucose, suppression of hepatic glucose production, impaired insulin signaling pathways, and release of proinflammatory adipokines [4, 14, 15]. Recent studies have demonstrated that this hyperinsulinemia-IR-obesity nexus is involved in the pathogenesis of T2D and the concomitant physiological and metabolic derangements increase the risk to the early progression of diabetes-associated complications, and predisposition towards hepatic, renal and cardiometabolic disorders [16–18].

Interestingly, emerging evidence indicates that individuals with normal weight might also be affected by hyperinsulinemia and IR [3, 19, 20]. Although the exact mechanisms contributing to the etiology and risk factors in normal-weight individuals are still unclear, it is hypothesized that central adiposity (that might not be reflected in the body mass index (BMI) calculations) in lean individuals can stimulate specific insulin-dependent pathways, triggering the manifestations of metabolic disturbances and accelerated systemic inflammation [21–23]. Overstimulation of visceral adipocyte size and depots, increased production of leptin and inflammatory cytokines such as tumor necrotic factor-α (TNF-α)) and interleukin-6 (IL-6) and lower expression of adiponectin in individuals with visceral adiposity have been documented [17, 24, 25], highlighting the critical role that visceral fat plays in releasing free fatty acids and pro-inflammatory markers that impair insulin signaling pathways and contribute to IR.

In epidemiological studies, the common methods to assess obesity include body mass index, as a measure of general obesity, and waist circumference, and waist-to-height ratio as measures of central obesity. Yet these measures are limited in terms of interpretations as they cannot estimate the body fat percent. A more detailed estimation of body composition indicators such as fat mass (FM), fat-free mass (FFM), and body fat percent will mitigate the limitation of using body measurements alone for obesity assessments, and adjusting FM and FFM values for an individual’s height might prove more clinically relevant [26]. Given that IR is typically associated with specific phenotypic characteristics (such as increased body weight and body fat, abdominal adiposity, and low muscle mass), a comprehensive assessment of overall and central adiposity and body composition measures will help identify the at-risk population and potential screening targets for diabetes and other future metabolic disorders.

In recent years, India has witnessed a sharp rise in obesity rates accompanied by an increasing burden of diabetes, cardiovascular diseases, and other metabolic disorders in youth [4, 11, 15, 27, 28]. Asian Indians are inherently predisposed to IR compared to their Caucasian counterparts, a phenomenon exacerbated by their unique “thin-fat phenotype,” characterized by higher visceral fat and body fat percentages despite having a lower BMI [16, 29]. This predisposition combined with a shift from traditional diets to energy-dense, nutrient-poor choices and reduced physical activity, as reported in several studies [30–32], increases the risk of Indians developing IR at a younger age and lower BMI thresholds, underscoring the need for early detection and targeted interventions. A few studies have investigated obesity and prediabetes prevalence in Indian youth [4, 5, 25]. However, there remains a significant gap in community-based research examining the prevalence and clinical correlates of hyperinsulinemia, an early indicator of metabolic dysfunction. To address this gap, the primary aim of our investigation was to determine the prevalence of hyperinsulinemia and insulin resistance in 16–25-year-old adolescents and young adults and evaluate their associations with measures of general and central adiposity and body composition. The analysis included participants who were screened for participation in our clinical trial assessing the effects of almond consumption on glycemic control and cardiometabolic health markers [33].

## Methods

### Design, setting, and participants

This cross-sectional study was conducted among a sample of 1313 adolescents and young adults, ages 16–25 years in Mumbai, India. The sampling protocol is described in our previous publication [33]. In summary, for participant recruitment, we approached twenty-four academic institutions (six junior colleges providing higher secondary education to 16-17-year-old adolescents and eighteen senior colleges imparting post-secondary higher education to 18-25-year-old young adults). Of these institutes, eleven institutes (junior colleges (*n* = 3) and senior colleges (*n* = 8) that provided permission were selected as the study sites. A junior college is an institute that offers two years of postsecondary education either as complete training or in preparation for admission to a degree program at a university in India. For our study, we randomly selected a class each from first- and second-year junior college (ages 16–17 years), the first, second, and third years of graduation programs in senior colleges (ages 18–21 years), and the first and second years of postgraduation degree and doctoral programs (22–25 years) as the sampling frame. All students attending these classes were verbally explained the study protocol and informed assent and parent consent forms (for participants < 18 years), and informed consent forms for those ≥ 18 years were distributed. The inclusion criteria were adolescents and young adults in the age group of 16–25 years, and providing written parent consent and written informed assent and/ or consent. Individuals with known chronic diseases, in the state of pregnancy and lactation, and on prescribed medications such as steroids were not included. Assuming a prevalence of hyperinsulinemia of 30% (as reported in previous studies conducted outside India among adolescents and youth [34, 35]), a margin of error of 5% and a confidence level of 99%, the estimated sample size was calculated as 560. To ensure adequate representation of both adolescent and young adult groups and account for an anticipated non-response rate of approximately 20%, the final sample size was estimated as 1344. The final analysis was performed for 1,313 participants (457 men and 856 women), who completed the survey and all biochemical assessments.

The study protocol and procedures were approved by an independent ethics committee, the Intersystem Biomedical Ethics Committee, Mumbai, India (ISBEC version 2 dated 12th Aug, 2017) before the start of the data collection. This study was conducted according to the guidelines laid out in the Declaration of Helsinki and Good Clinical Practices.

### Survey instrument

Participants completed a brief questionnaire including socio-demographic characteristics, family history of diabetes and hypertension (first- and second-degree relatives), and medical history of diabetes, hypertension, and hormonal disorders (such as thyroid disorders and polycystic ovarian syndrome). Participants were also asked to report the frequency with which they engaged in at least 30 min of walking or other moderate-intensity exercise on five or more days per week to determine whether they met the minimum recommended activity levels.

### Biochemical assessments and classification criteria

Fasting plasma glucose and insulin, 2-hour post glucose (75 g of glucose was administered) challenged plasma glucose, and stimulated insulin and glycosylated hemoglobin (HbA1C) were assayed by trained and qualified phlebotomists at the study sites using standard procedures. The serum samples were transported in ice boxes to the laboratory and stored at minus 80 degrees before analyses. Fasting blood glucose and 2 h post glucose blood glucose levels were estimated using the colorimetric method of Glucose Oxidase- Peroxidase or GOD-POD (Accurex Biomedical Pvt Ltd) and the fasting and stimulated insulin were measured by the immunoassay method (Beckman Coulter Counter). Glycosylated hemoglobin was measured using the Nycocard reader (Alere Technologies, Norway). As per the ADA (2017) criteria, participants having fasting insulin ≥ 15mIU/ml and/or stimulated insulin ≥ 80mIU/ml were classified as having hyperinsulinemia, and those with fasting glucose 5.6-6.9mmol/L and/or 2-hour post blood glucose between 7.8 and 11.0 mmol/L as having hyperglycemia [36]. The diagnosis of prediabetes was confirmed using either impaired fasting glucose (IFG) or impaired glucose tolerance (IGT) or both.

We used the Homeostasis Assessment Model (HOMA), Quantitative Insulin Sensitivity Check Index (QUICKI), and Fasting Glucose: Fasting Insulin (FG: FI) ratio to determine the prevalence of IR in participants [37]. HOMA-IR was calculated using the formula HOMA-IR = fasting insulin (µIU/ml) × fasting glucose (mmol/ml)/ 22.5 or HOMA-IR = fasting insulin (mg/dl) × fasting glucose (mg/dl)/ 405 [37, 38]. QUICKI was computed as 1/[Log (Fasting Insulin, µU/ml) + Log (Fasting Glucose, mg/dl)], and the FG: FI ratio was calculated by dividing the fasting glucose by fasting insulin values. Given the lacunae of population-based studies that have established a specific cut-off of HOMA-IR in the Indian population, and to ensure a comparison of our data with similar studies conducted globally, we used the widely used cut-off of HOMA_IR > 2.5 as indicative of insulin resistance [9, 21]. Participants were considered as IR when QUICKI ≤ 0.33 and FG: FI < 4.5 [37].

### Physical examination and anthropometry measurements

Blood pressure was checked by the study physician and the anthropometric measurements of weight, height, waist circumference (WC) and hip circumference (HC) were taken by research assistants having postgraduate degrees in public health and nutrition. Weight was measured without shoes to the nearest 0.1 kg on an electronic weighing scale (Equinox, Model EB6171), the height was taken on a stadiometer to the nearest centimeter (cm) and the WC and HC were measured using a non-stretchable, non-elastic measuring tape (model 205, Seca, Germany) over light clothing as per the standard procedures. For participants > 18 years, the WHO Asian criteria of body mass index 23–24.9 kg/m^2^ and ≥ 25 kg/m^2^ were used to estimate the prevalence of overweight and obesity respectively [39]. As per the AAC/AHA 2017 guidelines, hypertension was defined as systolic BP (SBP) ≥ 130 mm Hg or diastolic BP (DBP) ≥ 80 mm Hg [40]. The WC > 80 cm (women) and WC > 85 cm (men) were used as measures of central obesity. For adolescents, ages 16–17 years, the sex-specific BMI for age percentiles > 85th was classified as overweight and > 90th as obese, WC > 90th percentile as a measure of central adiposity, and SBP/ DBP > 95th percentile for age as the presence of hypertension [11, 40]. Similar to previous studies, the WHtR values > 0.5 were interpreted as a measure of central adiposity in participants [41].

### Body composition

The body composition was measured using a multifrequency 8-electrode bioelectric impedance analyzing body composition machine (Tanita MC 780 MA, TANITA Corporation, Japan, minimum calibration 0.1 kg) by trained field investigators. For greater accuracy, the investigators ensured that all measurements were taken in the morning before participants had consumed meals, or fluids, or had exercised. The body fat and visceral fat percent, and fat and fat-free mass (in kg) were recorded in duplicate and then averaged for each participant. We defined obesity as body fat ≥ 25% in men and ≥ 35% in women, and overweight as ≥ 20% in men and ≥ 30% in women [41]. Additionally, to adjust fat and fat-free mass for height, we calculated the Fat Mass Index (FMI) and Fat-Free Mass Index (FFMI) using the formula- FM or FFM (kg) divided by height in m^2^. FMI is a simple tool that can be used in addition to BMI to identify individuals who may have normal BMI but elevated fat mass and to correct the body fat percent values for variations in height of participants in large-scale population studies [26].

### Statistical analysis

All analyses were performed using the statistical software package, STATA (14.2). Descriptive statistics (mean (standard deviation) median (interquartile range), number (percentage)) were used to describe the sociodemographic and anthropometric characteristics and clinical profile of the participants. Associations at the univariate level were assessed by employing an independent Student’s t-test and analysis of variance (ANOVA) depending on the categories of variables involved. Logistic regression analyses determined the sex-adjusted effects of independent variables (age, family history, lifestyle factors, anthropometric measurements, and blood pressure) on hyperglycemia (elevated fasting and/or 2 h post glucose challenge blood glucose levels) and hyperinsulinemia (elevated fasting and/or 2 h stimulated insulin levels) in separate models. The odds ratios (ORs) and their 95% confidence intervals (CI) were calculated and a two-sided *p* ≤ 0.05 was considered a measure of statistical significance.

## Results

### Participant characteristics

Data from eligible adolescents, ages 16–17 years (*n* = 446) who provided informed written assent and parental consent, and 18–25-year-old young adults (*n* = 867) with written informed consent to participate, were analyzed. Of 1313 participants (mean age 19.6 (2.1) years), 65.2% were females, 55.1% belonged to the age group of 16–19 years and 12.4% had both first and second-degree relatives with diabetes. A total of 610 participants (46.4%) reported having no family history of diabetes. A known history of elevated blood pressure or hormonal disorders was reported by 5.9% and only half (52.3%) were engaging in > 2.5 h/ week of physical activity (Table 1).

**Table 1:** Demographic characteristics and body weight status of participants (*n* = 1313)

Characteristics	*n* (%)
**Gender**	
Males Females	457 (34.8) 856 (65.2)
**Age Categories**	
16–19 years 20–22 years 23–25 years	724 (55.1) 495 (37.7) 94 (7.2)
**Family history of diabetes**	
First degree family member (parents/siblings) Second degree family member (grandparents/ uncle/aunts)	132 (10.1) 408 (31.1)
Both first- and second-degree family members No family member with diabetes	163 (12.4) 610 (46.4)
**Medical history**	
Known history of elevated blood pressure Hormonal disorders (PCOS/ thyroid disorders)	19 (1.5) 58 (4.4)
**Activity pattern**	
Physical activity > 2.5 h/week (Yes)	685 (52.3)
**Body weight status (n = 1310)** ^**#**^	
Underweight Normal weight Overweight Obese	310 (23.7) 535 (40.8) 197 (15.0) 268 (20.5)
**Central adiposity measure (n = 1310)**	
Waist to height ratio > 0.5	280 (21.3)

^#^Participants ≥ 18 years- Classification based on WHO Criteria for Asians (2004)

Participants < 18 years-Classification based on BMI- For-Age Growth Charts (CDC, 2022)

### Prevalence of hyperinsulinemia, insulin resistance and obesity

As per the ADA criteria of either impaired fasting glucose (2.1%) or impaired glucose tolerance (2.4%) or both, the prevalence of prediabetes was 4.5%. Elevated fasting insulin (≥ 15 mIU/ml) and 2 h stimulated insulin (≥ 80 mIU/ml) were observed in 9.0% and 30.5% respectively. The combined prevalence of overweight and obesity was 35.5% and abdominal obesity (WHtR > 0.5) was 21.3%. The presence of abdominal obesity was observed in participants with BMI ≥ 25 kg/m^2^ (34.6%) but also in those with BMI ≤ 22.9 kg/m^2^ (15.1%). Overall, 5.3% had an FG: FI ratio < 4.5, 4.4% were hypertensive and 9.9% had HbA1c levels between 5.7% and 6.4% (indicative of prediabetes) (Table 2).

**Table 2:** Prevalence of hyperinsulinemia, insulin resistance, obesity, and hypertension among 16-25-year-old adolescents and young adults

Parameters	Total (*n* = 1313)	Adolescents 16–19 years (*n* = 724)	Young Adults 20–25 years (*n* = 589)	*p*-value
Fasting insulin				
Normal (< 15mIU/ml)	1195 (91.1)	668 (92.3)	527 (89.5)	0.042^*^
Elevated (≥ 15 mIU/ml)	118 (9.0)	56 (7.7)	62 (10.5)	
Stimulated insulin				
Normal (< 80mIU/ml)	913 (69.5)	589 (81.4)	324 (55.1)	0.053
Elevated (≥ 80 mIU/ml)	400 (30.5)	135 (18.6)	265 (44.9)	
Fasting glucose				
Normal (< 100 mg/dL)	1285 (97.9)	711 (98.2	574 (97.5)	0.405
Elevated (100- 125 mg/dL)	28 (2.1)	13 (1.8)	15 (2.5)	
2 -hour post glucose				
Normal (< 140 mg/dl)	1282 (97.6)	712 (98.3)	570 (96.8)	0.981
Elevated (140–199 mg/dL)	31 (2.4)	12 (1.7)	19 (3.2)	
HbA1C (< 5.7%) * HbA1c (5.7–6.4%) HbA1c (≥ 6.5%)	533 (40.6) 131 (9.9) 3 (0.3)	235 (32.5) 42 (5.8) 1 (0.13)	298 (50.6) 89 (15.1) 2 (0.34)	< 0.001^***^
HOMA IR > 2.5	227 (17.3)	113 (15.6)	114 (19.4)	0.543
FG: FI ratio < 4.5	69 (5.3)	38 (5.3)	31 (5.3)	0.862
QUICKI < 0.33	199 (15.2)	114 (15.8)	85 (14.4)	0.722
SBP > 130 mmHg DBP > 80 mmHg	46 (3.5) 12 (0.9)	24 (3.3) 5 (0.7)	22 (3.7) 7 (1.2)	0.585 0.311
BMI ≤ 22.9 kg/m^2^ BMI 23–24.9 kg/m^2^ BMI > 24.9 kg/m^2^	844 (64.3) 200 (15.2) 269 (20.5)	513 (70.8) 101 (13.9) 110 (15.2)	331 (56.2) 99 (16.8) 159 (26.9)	< 0.001^***^
Waist to height ratio > 0.5	280 (21.3)	113 (15.6)	167 (28.3)	< 0.001^***^

*n = 667; ^***^p < 0.001

HbA1c, Glycosylated Hemoglobin; HOMA-IR, Homeostasis Assessment Model- Insulin Resistance

FG: FI, Fasting Glucose: Fasting Insulin; QUICKI, Quantitative Insulin Sensitivity Check Index

SBP, Systolic Blood Pressure, DBP, Diastolic Blood Pressure, BMI, Body Mass Index

BMI > 22.9 kg/m^2^ indicative of overweight, and BMI > 24.9 kg/m^2^ of obesity (WHO Asian Criteria)

Waist to height ratio > 0.5 indicates central obesity

Based on HbA1C values, the prevalence of prediabetes was 15.1% in young adults ≥ 20 years and 5.8% in 16-19-year-old adolescents (*p* < 0.001). Similarly, the prevalence of overweight (16.6% Vs 13.9%), obesity (26.9% Vs 15.2%), and central obesity (WHtR as 28.3% Vs 15.6%) was higher among participants ≥ 20 years than those < 20 years (*p* < 0.001). No differences were observed in IFG, IGT, hyperinsulinemia, and hypertension prevalence rates between adolescents and young adults.

Comparison of variables between girls and boys is provided in Supplementary Table 3.

**Table 3:** Comparison of variables between participants having different body weight status

Variables	Overall (*n* = 1313)	Underweight (*n* = 306)	Normal Weight (*n* = 538)	Overweight (*n* = 200)	Obese (*n* = 269)	*p*-value
Waist-to-hip ratio	0.78 (0.06)	0.76 (0.05)	0.77 (0.05)	0.79 (0.06)	0.82 (0.07)	< 0.001^***^
Waist-to-height ratio	0.45 (0.06)	0.39 (0.03)	0.43 (0.03)	0.47 (0.03)	0.53(0.05)	< 0.001^***^
Body fat %	26.1 (8.9)	18.4 (6.3)	24.5 (6.5)	29.3 (6.1)	35.5 (7.5)	< 0.001^***^
Visceral Fat	4.1 (2.1)	1.1 (0.5)	2.9 (1.4)	5.6 (1.5)	8.5 (2.5)	< 0.001^***^
SBP	105.1 (11.4)	101.3 (10.2)	104.2 (10.8)	106.8 (11.3)	109.8 (12.2)	< 0.001^***^
DBP	66.7 (8.1)	64.6 (7.6)	66.1 (7.7)	67.6 (7.9)	69.8 (8.6)	< 0.001^***^
FBG	82.1 (10.5)	81.1 (8.3)	80.8 (7.9)	83.3 (10.6)	84.5 (15.7)	< 0.001^***^
PBG	93.3 (25.1)	88.1 (17.7)	91.6(22.9)	96.1 (25.5)	100.8 (33.3)	< 0.001^***^
HbA1c	5.4 (0.4)	5.4 (0.3)	5.4 (0.3)	5.4 (0.4)	5.5 (0.5)	0.111
Fasting Insulin	8.7 (5.5)	6.8 (4.8)	7.9 (4.1)	9.9 (6.7)	11.7 (6.3)	< 0.001^***^
Stimulated Insulin	72.6 (31.3)	59.6 (27.6)	69.3 (34.4)	77.1 (27.1)	90.6 (68.1)	< 0.001^***^
FG: FI	12.1 (6.5)	14.7 (6.6)	12.4 (6.4)	10.8 (6.0)	8.9 (5.4)	< 0.001^***^
HOMA IR	1.8 (1.3)	1.4 (0.9)	1.6 (0.9)	2.1 (1.2)	2.4 (1.4)	< 0.001^***^
QUICKI	0.35 (0.05)	0.37 (0.04)	0.36 (0.05)	0.35 (0.04)	0.33 (0.05)	< 0.001^***^

Data is presented as mean (SD); ^***^p < 0.001

SBP, Systolic Blood Pressure (mmHg); DBP, Diastolic Blood Pressure (mmHg)

FBG, Fasting Blood Glucose (mg/dL); PBG, Post glucose Blood Glucose (mg/dL)

HbA1c, Glycosylated Hemoglobin

FG: FI, Fasting Glucose: Fasting Insulin; HOMA IR, Homeostatic Model Assessment of Insulin Resistance

QUICKI, Quantitative Insulin Sensitivity Check Index

### Biochemical parameters and body composition measures of participants with different body weight status

Participants with overweight and obesity had significantly greater mean body fat percent, visceral fat, and systolic and diastolic blood pressure values as compared to those with BMI ≤ 22.9 kg/m^2^. Significant differences were observed in mean blood glucose and insulin levels and IR parameters such as HOMA-IR, FG: FI ratio, and QUICKI between participants having varying BMI status (Table 3). The prevalence of hyperinsulinemia was significantly higher in participants with overweight or obesity as compared to those with normal weight (*p* < 0.001). Insulin resistance was observed in 17.3% with a higher prevalence in those with BMI > 22.9 kg/m^2^ (32.4%) as compared to those with BMI ≤ 22.9 kg/m^2^ (8.9%, *p* < 0.001). Interestingly, of all participants who had IR (*n* = 227), almost one-third (33.1%) were either underweight or normal weight. More than half of the participants with prediabetes (58.1%) were overweight compared to 29.9% of those with normoglycemia. Additionally, central adiposity was prevalent among participants having prediabetes, with 25.0% having a WC above sex-specific cut-offs and 29.1% having a WHtR greater than 0.5.

### Comparison of variables between participants with and without hyperinsulinemia and hyperglycemia

Based on the blood glucose and insulin levels, the participants were categorized into four categories: (1) normoglycemia and normal-insulinemia (*n* = 841, 64.1%) (2) hyperinsulinemia but normoglycemia (*n* = 395, 30.1%), (3) hyperglycemia but normal-insulinemia (*n* = 16, 1.2%), and (4) hyperinsulinemia and hyperglycemia (*n* = 37, 2.8%). Comparison of variables between groups indicated significantly higher mean weight, BMI, waist and hip circumferences, body composition indices (body fat % and visceral fat), and systolic and diastolic blood pressure values in participants with hyperglycemia and hyperinsulinemia (category 4) as compared to those with normoglycemia and normal insulinemia (category 1) (Table 4).

**Table 4:** Comparison of variables between participants with and without hyperinsulinemia and hyperglycemia

Variables	Category 1 *n* = 841	Category 2 *n* = 395	Category 3 *n* = 16	Category 4 *n* = 37	*p*-value
Weight (kg)	55.5 (12.3)	57.9 (14.3)	52.2 (12.9)	62.5 (9.5)	0.002^**^
BMI (kg/m^2)^	21.4 (4.4)	23.0 (4.7)	19.8 (3.9)	25.4 (4.5)	< 0.001^***^
Waist circumference (cm)	70.2 (9.7)	73.6 (11.1)	66.4 (7.7)	76.6 (8.5)	< 0.001^***^
Hip circumference (cm)	89.9 (9.5)	93.1(10.3)	85.9 (8.5)	96.8 (8.3)	< 0.001^***^
Waist-to-Height Ratio	0.4 (0.05)	0.4 (0.06)	0.4 (0.04)	0.4 (0.06)	< 0.001^***^
Body fat (%)	24.2 (8.8)	29.4 (7.8)	23.1 (7.4)	32.3 (9.3)	< 0.001^***^
Visceral Fat (%)	3.8 (3.06)	4.6 (3.3)	3.2 (2.7)	5.9 (2.6)	< 0.001^***^
Fat Mass Index	9.6 (4.1)	11.9 (3.5)	14.5 (1.3)	12.3 (4.7)	< 0.001^***^
Fat-free Mass Index	13.2 (5.4)	13.8 (4.6)	15.2 (0.8)	14.9 (4.3)	0.021^*^
HbA1c	5.4 (0.3)	5.4 (0.3)	5.4 (0.2)	5.5 (0.3)	0.520
Diastolic blood pressure (mmHg)	66.4 (8.1)	66.9 (8.0)	66.9 (8.0)	71.6 (9.1)	0.002^**^
Systolic blood pressure (mmHg)	104.8 (11.2)	104.89 (11.7)	106.4 ((11.9)	111.3 (11.1)	0.007^**^

^*^p < 0.05, ^**^p < 0.01, ^***^p < 0.001

BMI, Body Mass Index. HbA1C, Glycosylated Hemoglobin

Category 1: normoglycemic and normoinsulinemia; Category 2: hyperinsulinemia but normoglycemia

Category 3: hyperglycemia but normoinsulinemia; Category 4: hyperinsulinemia and hyperglycemia

### Factors associated with the presence of hyperglycemia and hyperinsulinemia

In the unadjusted regression models, the odds of hyperglycemia were higher in participants with overweight (OR 2.52 (95% CI, 1.95, 6.70) *p* = 0.03) and obesity (OR 4.92 (95% CI, 1.40, 7.24), *p* = 0.01) (Table 5). The presence of hyperinsulinemia was observed to be associated with body fat percent (OR 1.27 (95% CI, 1.15,1 0.40) *p* < 0.001) and waist-to-height ratio (95% CI, 1.70(1.10, 3.45), *p* = 0.02). The odds of hyperglycemia were 2.52 times higher in individuals with overweight (95% CI 1.95, 6.70, *p* = 0.03) and 4.92 times in those with obesity (95% CI 1.40, 7.24) *p* = 0.01). In sex-adjusted models, hyperinsulinemia showed significant associations with body fat percent (OR 1.13 (95% CI 1.02, 1.29) *p* = 0.006), WHtR (OR 1.66 (95% CI,1.15, 3.30), *p* = 0.02) and blood pressure (OR 1.08, 95% CI 1,03, 1.20, *p* = 0.04).

**Table 5:** Association of various variables with hyperglycemia and hyperinsulinemia in study participants (*n* = 1313), adjusted and unadjusted by sex

Variables	Factors associated with hyperglycemia (*n* = 59) ^#^				Factors associated with hyperinsulinemia (*n* = 518) ^#^			
	Unadjusted OR (95%CI)	*p*-value	Sex adjusted OR (95%CI)	*p*-value	Unadjusted OR (95%CI)	*p* - value	Sex adjusted OR (95%CI)	*p* - value
Age	0.98 (0.84,1.14)	0.80	0.99 (0.85, 1.15)	0.93	0.99 (0.92, 1.05)	0.76	0.99 (0.92, 1.05)	0.76
First-degree relative with diabetes	1.19 (0.62, 2.27)	0.59	1.18 (0.61, 2.24)	0.61	1.29 (0.96, 1.74)	0.09	1.29 (0.96, 1.74)	0.09
Second-degree relative with diabetes	1.13 (0.63, 2.03)	0.67	1.14 (0.63, 2.06)	0.64	0.81(0.62, 1.06)	0.13	0.81 (0.62, 1.06)	0.13
Physical activity	0.92 (0.73, 2.90)	0.37	0.90 (0.68, 2.16)	0.33	0.91(0.82, 1.23)	0.32	0.93 (0.88, 1.19)	0.21
Body fat (%)	1.02 (0.97,1.07)	0.36	1.08 (0.96, 1.21)	0.15	**1.27 (1.15**,** 1.40)**	**< 0.001** ^***^	**1.13 (1.02**,** 1.29)**	**0.006** ^**^
Visceral fat	0.94 (0.78, 1.13)	0.53	0.84(0.65, 1.10)	0.21	0.97(0.89, 1.05)	0.54	0.97 (0.87, 1.09)	0.68
Waist-to-Height Ratio	1.10 (0.90, 1.37)	0.16	1.01 (0.86, 1.25)	0.17	**1.70 (1.10**,** 3.45)**	**0.02**	**1.66 (1.15**,** 3.30)**	**0.02**
Overweight	**2.52 (1.95**,** 6.70)**	**0.03** ^*^	**2.41(1.91**,** 6.41)**	**0.03** ^*^	0.91 (0.68, 1.40)	0.67	0.90 (0.79,1.43)	0.68
Obese	**4.92 (1.40**,** 7.24)**	**0.01** ^**^	**4.67 (1.33**,** 6.37)**	**0.01** ^**^	0.76 (0.42, 1.36)	0.36	0.76(0.42, 1.37)	0.37
Blood Pressure	0.99 (0.90–1.10)	0.66	0.99 (0.91–1.10)	0.61	**1.11 (1.04–1.20)**	**0.04** ^*^	**1.08 (1.03–1.20)**	**0.04** ^*^

OR, Odds Ratio. CI, Confidence Interval. ^*^p < 0.05, ^**^p < 0.01, ^***^p < 0.001

^#^Hyperglycemia - impaired fasting and/or impaired glucose tolerance. ^##^Hyperinsulinemia- elevated fasting and/or 2 h stimulated insulin

Reference: Age < 20 years; Physical activity < 2.5 h/week; Body fat % < 20% in men and < 30% in women; Waist to Height Ratio < 0.5

Reference- Normal/Underweight (BMI < 22.9 kg/m^2^) for variables- overweight and obese

Reference: Blood Pressure – Systolic Blood Pressure < 130mmHg and/or Diastolic Blood Pressure < 80 mmHg

## Discussion

Hyperinsulinemia and insulin resistance (IR) are early indicators of metabolic dysfunction that often precede the development of conditions such as T2D, hypertension, and cardiovascular diseases. Identifying these markers in apparently healthy youth—who may not yet exhibit clinical symptoms—is essential for preventing disease progression and minimizing long-term healthcare burdens. IR is closely associated with obesity, a condition that has reached epidemic levels globall**y**, including in India. However, despite the increasing prevalence of obesity in Indian youth, data on the prevalence of IR and hyperinsulinemia in this demographic remain limited. Furthermore, there is a lack of comprehensive studies examining the association of obesity—measured through indicators such as body mass index (BMI), body fat percentage, and markers of abdominal adiposity (e.g., waist circumference and waist-to-height ratio)—with IR and hyperinsulinemia. Our study explored the interplay between obesity and metabolic dysfunction in Indian youth to provide preliminary evidence, crucial for developing tailored screening and intervention strategies to improve long-term health outcomes.

The key findings of the study included (1) a substantially high prevalence of hyperinsulinemia (39.7%), insulin resistance (17.3%), and overweight and obesity (35.5%) in the sampled young population, (2) presence of abdominal obesity in participants with general obesity (34.6%) but also in those with normal weight (15.1%) (3) prediabetes prevalence as 4.5% with more than half (58.1%) being overweight and almost one-third (29.1%) having abdominal obesity, (4) a greater prevalence of HbA1C determined prediabetes in 20-25-year-old young adults (15.1%) compared to adolescents (5.8%), (5) higher mean values of blood pressure, blood glucose and insulin, and IR indicators such as HOMA-IR, QUICKI and FG: FI ratio in participants having BMI ≥ 22.9 kg/m^2^ than those with BMI < 22.9 kg/m^2^, and (6) Increasing odds of hyperglycemia and hyperinsulinemia with increases in BMI, body fat percent, and abdominal obesity (WHtR > 0.5) in sex-adjusted regression models.

Due to the paucity of hyperinsulinemia and IR prevalence data in youth in India as elsewhere, we compared our findings with a few studies that were conducted either among children and adolescents or had reported prevalence of prediabetes, diabetes, and IR in middle-aged adults. For instance, a recent school-based study in eastern India among 6-16-year-old children who were overweight or obese reported the prevalence of IR as 32.3% and an increased risk of IR in those with increased WC and blood pressure [11]. A secondary analysis of data collected as a part of the Comprehensive National Nutrition Survey (CNNS) of India reported prediabetes and diabetes prevalence in adolescents ages 10–19 years as 16.2% and 0.56% respectively [1]. Another analysis of India’s National Family Health Survey-4 (NFHS-4) data observed the prevalence of diabetes at 3.75% and prediabetes at 4.56% in adults aged 18–25 years with positive associations between diabetes and urban residence, age, and body mass index [4]. Concurrent with these findings, we observed that the prevalence of prediabetes was 4.5% based on IFG, and/or IGT, and 9.9% based on HbA1C values. The presence of hyperinsulinemia and IR at a younger age is reported to be marked by an aggressive progression and early onset of diabetes [15, 42, 43]. This presents a serious personal and public health consequence as longer exposure to IR and its associated metabolic abnormalities is likely to escalate the risk of microvascular complications, contributing to a greater economic burden and poor quality of life, as adolescents and young adults transition into adulthood.

The time trend studies conducted outside India highlight the increasing global incidence of T2D in adolescents and young adults. According to the most recent international data from the SEARCH study published in 2023, the overall adjusted incidence of T2D in children and adolescents has increased exponentially (two to threefold) from 2002 to 2018 [44]. Similarly, data regarding youth-onset diabetes from China revealed an annual increase of 26.6% among 10–19-year-old young adults with the risk being almost 1.5 times higher in urban areas as compared to rural settings [15]. In the US, a pooled analysis of NHANES data (2015-18 cycle) reported the prevalence of IR in 18-44-year-old young adults as 40.3% and significant associations between HOMA-IR, BMI, waist circumference, and hypertension [19]. A systematic review and meta-analysis of 48 studies reported the global prevalence of childhood prediabetes as 8.84% with a significant upward trend over the last decade, parallel to the increase in the frequency and severity of childhood obesity prevalence [44]. Data from long-term studies such as the TODAY (Treatment Options for T2D in Adolescents and Youth) [42] and the RISE (Restoring Insulin Secretion) trial [45] suggest a direct association between insulin resistance and a rapid progression of beta cell dysfunction and a higher incidence of complications such as arterial stiffness, hypertension, and dyslipidemia in those having youth-onset T2D. These findings reiterate the importance of detecting hyperinsulinemia in adolescents and young adults as a window of opportunity for primordial and primary prevention of adverse health outcomes in the future.

In addition to the prevalence data, we investigated the associations between prediabetes, IR, and obesity in adolescents and young adults. Several anthropometric and body composition measures were determined to explore whether these adiposity measures varied across the spectrum of normoglycemia and normoinsulinemia to hyperglycemia and hyperinsulinemia. The results indicated that participants with hyperinsulinemia and IR were more likely to be overweight and had higher waist circumference, WHR, and WHtR. These findings concur with various cross-sectional studies showing that central obesity is strongly linked to elevated fasting insulin levels and HOMA-IR, key markers of IR [1, 2, 17]. A remarkable finding was that 30.1% of participants with a BMI < 22.9 kg/m^2^ (normal and/ or underweight) had hyperinsulinemia. Additionally, 15.1% of participants with normal weight had abdominal obesity, and one-third of those with IR were neither overweight nor obese. While BMI-defined obesity is often regarded as an early clinical indicator of IR and is used to guide prevention and intervention strategies, our findings suggest that excess body weight alone may not adequately capture metabolic risk in adolescents and young adults. Several factors may explain the observed IR in partcipants having normal weight, including genetic predisposition, visceral adiposity despite normal BMI, early life nutritional exposures, sedentary behavior, and population-specific differences in fat distribution and insulin sensitivity. The finding underscores the importance of broader assessment beyond BMI, including WC, WHR, and WHtR, along with body composition metrics like body fat percentage, fat-free mass, and visceral fat. Early identification of metabolically at-risk individuals and screening for insulin resistance and hyperinsulinemia, even among individuals with normal body weight, merits further research and could provide a crucial opportunity to prevent and manage future cardiometabolic diseases.

In our study, adolescents and young adults with overweight/ obesity and those with hyperinsulinemia and hyperglycemia had higher systolic and diastolic blood pressure values compared to those with normal weight and normal glucose and insulin levels. The close association of obesity with hypertension is well-documented [19, 46]. Obesity leads to chronic inflammation and oxidative stress, contributing to vascular stiffness and endothelial dysfunction. Excess adiposity is also known to trigger overactivity of the sympathetic nervous system (SNS) and hyperactivation of the renin-angiotensin-aldosterone system (RAAS), further elevating blood pressure [47, 48]. In recent years, there has been an emerging interest in understanding the common pathways that can explain the link between hypertension, insulin resistance, and resultant hyperinsulinemia. Obesity-linked insulin resistance plays a key role in the pathogenesis and progression of hypertension, primarily due to the pleiotropic effects that insulin exerts on lipid and protein metabolism, neurohormonal activities, and regulation of vascular tone, function, and inflammation [48–50]. Independent of obesity, hyperinsulinemia can contribute to adipokine dysregulation (increased leptin levels and reduced adiponectin) and reduced NO bioavailability, mechanisms that are implicated in vasoconstriction and increased peripheral vascular resistance [8, 17]. The multifaceted molecular and pathophysiological mechanisms underlying hypertension and hyperinsulinemia along with the complex cluster of symptoms associated with the overlapping metabolic and vascular dysfunctions can escalate the risk of cardiovascular diseases in youth, warranting a strict control of both blood pressure and glycemia, irrespective of their body weight status.

There are several public health implications of this community-based cross-sectional study. First, the prevalence of hyperinsulinemia, IR, and prediabetes was estimated among a large sample of Indian adolescents and young adults, providing descriptive epidemiological evidence for relatively less commonly screened yet clinically significant indicators that precede the onset of frank diabetes. Second, the associations of hyperinsulinemia and IR with several concomitant general and central adiposity measures and body composition were evaluated. While WC and WHR indicated abdominal obesity, WHtR assessed the cardiometabolic risk of obesity and the BIA method of body composition measurement provided detailed insights into the pattern of body fat distribution and other components such as FFM and visceral fat, known to be linked with IR. Third, elevated blood pressure, visceral fat and body fat percentages were investigated as factors of hyperinsulinemia and hyperglycemia. Fourth, the sample comprised individuals in their post-pubertal period of late adolescence and young adulthood, an age group that has not been provided the required attention in targeted lifestyle intervention studies or public health policies. Finally, the anthropometric measurements, glycosylated hemoglobin, and blood pressure values were compared between participants with different combinations of normal and elevated glucose and insulin levels. These findings highlighted a clustering of cardiometabolic risk factors in adolescents and young adults with prediabetes and IR, suggesting that metabolic dysfunction begins early and may progress silently even before overt hyperglycemia develops. However, as this study was cross-sectional, longitudinal data would be needed to establish the temporal and predictive value of these associations in determining future risk of type 2 diabetes and cardiovascular disease. Such data would help clarify whether early hyperinsulinemia and insulin resistance serve as reliable predictors of long-term metabolic deterioration, thereby strengthening the evidence base for early screening and targeted intervention strategies in this age group. The insulin and glucose levels were measured in fasting and at 2 h post-glucose challenge states for all participants. Future research may consider using composite measures of beta cell functions, such as oral disposition index to evaluate how effectively beta cells respond to glucose **i**n relation to changes in insulin sensitivity.

The results should however, be interpreted in light of certain limitations. The study’s cross-sectional design restricts the ability to establish causal relationships and directionality of associations between hyperinsulinemia, IR, and obesity. Additionally, the sample was drawn from urban populations, which may limit the generalizability of the results to rural settings. Given the large sample size and the inclusion of multiple survey variables and biochemical assessments, we limited the questionnaire to include demographic characteristics and a single physical activity item to minimize respondent burden and fatigue. Also, the pubertal stage can influence metabolic parameters in adolescents. Hence, future research may consider assessing pubertal status in both boys and girls to enable more accurate comparisons. While lifestyle, diet, and physical activity-related questionnaires were administered during the trial phase of the larger study, these assessments were not performed for participants during the screening phase.

## Conclusions

A substantially high prevalence of hyperinsulinemia, and IR, concomitant with the presence of general and central obesity and high blood pressure, as observed in our study highlighted a clustering of cardiometabolic risk factors in the adolescent and young adult population. Current diabetes screening and management guidelines for adolescents and adults do not account for hyperinsulinemia and insulin resistance. Youth, aged 15 to 29 years, represent approximately one-third of the total population in India. This large and growing demographic is also at the forefront of the ongoing epidemiological and nutrition transition that is fueling the burden of health challenges like obesity, diabetes, and related metabolic disorders. Prediabetes in youth is linked to a more aggressive clinical progression, and IR is associated with early vascular dysfunction, so it is plausible that microvascular and subclinical changes may begin in adolescents and young adults with hyperinsulinemia and IR before diabetes becomes clinically apparent. Moreover, our findings related to the presence of hyperinsulinemia and IR in youth with normal weight underscore the urgent need for early screening of cardiometabolic risk factors and the implementation of targeted interventions to prevent the progression to overt diabetes and cardiovascular diseases.

## Supplementary Information

Below is the link to the electronic supplementary material.

Supplementary Material 1

Supplementary Material 2

Supplementary Material 3

## Data Availability

All data generated and /or analyzed during the current study are included in the published article and its supplementary information files (Supplementary Files 1, 2 and 3).
