# Supplementary material for: Prevalence of hyperinsulinemia and its association with measures of adiposity and body composition in 16-25-year-old adolescents and young adults in Mumbai
Source: BMC Endocr Disord. 2026 Mar 13;26:117. doi: 10.1186/s12902-026-02230-0 (PMC13097659; doi:10.1186/s12902-026-02230-0)
Supplement: Supplementary file 1 — Supplementary Material 1 [file 12902_2026_2230_MOESM1_ESM.pdf]

Supplementary File 1:
Results of Chi-Square Tests for comparison of variables between age categories in the study

	Valid	
	N	Percent
sugarcat * AGECAT	1313	100.0%
twohrsugarcat * AGECAT	1313	100.0%
FatingInsulinCat * AGECAT	1313	100.0%
twohourInsulinCAT * AGECAT	1313	100.0%
HbA1cCAT * AGECAT	667	100.0%
BMIcat * AGECAT	1313	100.0%
bodyfatcat * AGECAT	1313	100.0%
HOMACAT * AGECAT	1313	100.0%
FGFIRATIOCAT * AGECAT	1313	100.0%
SBPCAT * AGECAT	1313	100.0%
DBPCAT * AGECAT	1313	100.0%
QUIKICAT * AGECAT	1313	100.0%

sugarcat * AGECAT

Chi-Square Tests

	Value	df	Asymp. Sig. (2-sided)	Exact Sig. (2-sided)	Exact Sig. (1-sided)
Pearson Chi-Square	.694[a]	1	.405		
Continuity Correction[b]	.406	1	.524		
Likelihood Ratio	.688	1	.407		
Fisher's Exact Test				.405	.261
Linear-by-Linear Association	.693	1	.405		
N of Valid Cases	1313				

a. 0 cells (0.0%) have expected count less than 5. The minimum expected count is 11.87.

b. Computed only for a 2x2 table

twohrsugarcat * AGECAT

Chi-Square Tests

	Value	df	Asymp. Sig. (2-sided)	Exact Sig. (2-sided)	Exact Sig. (1-sided)
Pearson Chi-Square	.001[a]	1	.981		
Continuity Correction[b]	0.000	1	1.000		
Likelihood Ratio	.001	1	.981		
Fisher's Exact Test				.981	.565
Linear-by-Linear Association	.001	1	.981		
N of Valid Cases	1283				

a. 0 cells (0.0%) have expected count less than 5. The minimum expected count is 14.07.

b. Computed only for a 2x2 table

FatingInsulinCat * AGECAT

Chi-Square Tests

	Value	df	Asymp. Sig. (2-sided)	Exact Sig. (2-sided)	Exact Sig. (1-sided)
Pearson Chi-Square	4.122[a]	1	.042		
Continuity Correction[b]	3.715	1	.054		
Likelihood Ratio	4.091	1	.043		
Fisher's Exact Test				.042	.027
Linear-by-Linear Association	4.119	1	.042		
N of Valid Cases	1278				

a. 0 cells (0.0%) have expected count less than 5. The minimum expected count is 46.14.

b. Computed only for a 2x2 table

twohourInsulinCAT * AGECAT

Chi-Square Tests

	Value	df	Asymp. Sig. (2-sided)	Exact Sig. (2-sided)	Exact Sig. (1-sided)
Pearson Chi-Square	3.744[a]	1	.053		
Continuity Correction[b]	3.509	1	.061		
Likelihood Ratio	3.733	1	.053		
Fisher's Exact Test				.057	.031
Linear-by-Linear Association	3.741	1	.053		
N of Valid Cases	1313				

a. 0 cells (0.0%) have expected count less than 5. The minimum expected count is 169.28.

b. Computed only for a 2x2 table

HbA1cCAT * AGECAT

Chi-Square Tests

	Value	df	Asymp. Sig. (2-sided)
Pearson Chi-Square	3.717[a]	2	.000
Likelihood Ratio	3.855	2	.000
Linear-by-Linear Association	3.417	1	.000
N of Valid Cases	667		

a. 2 cells (33.3%) have expected count less than 5. The minimum expected count is 1.16.

BMIcat * AGECAT

Chi-Square Tests

	Value	df	Asymp. Sig. (2-sided)
Pearson Chi-Square	38.328[a]	3	.000
Likelihood Ratio	38.609	3	.000
Linear-by-Linear Association	37.808	1	.000
N of Valid Cases	1313		

a. 0 cells (0.0%) have expected count less than 5. The minimum expected count is 85.20.

WHtRcat * AGECAT

Chi-Square Tests

	Value	df	Asymp. Sig. (2-sided)	Exact Sig. (2-sided)	Exact Sig. (1-sided)
Pearson Chi-Square	43.974[a]	1	.000		
Continuity Correction[b]	43.190	1	.000		
Likelihood Ratio	43.891	1	.000		
Fisher's Exact Test				.000	.000
Linear-by-Linear Association	43.940	1	.000		
N of Valid Cases	1291				

a. 0 cells (0.0%) have expected count less than 5. The minimum expected count is 190.17.

b. Computed only for a 2x2 table

HOMACAT * AGECAT

Chi-Square Tests

	Value	df	Asymp. Sig. (2-sided)	Exact Sig. (2-sided)	Exact Sig. (1-sided)
Pearson Chi-Square	.370[a]	1	.543		
Continuity Correction[b]	.283	1	.595		
Likelihood Ratio	.369	1	.543		
Fisher's Exact Test				.541	.297
Linear-by-Linear Association	.370	1	.543		
N of Valid Cases	1291				

a. 0 cells (0.0%) have expected count less than 5. The minimum expected count is 90.03.

b. Computed only for a 2x2 table

FGFIRATIOCAT * AGECAT

Chi-Square Tests

	Value	df	Asymp. Sig. (2-sided)	Exact Sig. (2-sided)	Exact Sig. (1-sided)
Pearson Chi-Square	.030[a]	1	.862		
Continuity Correction[b]	.002	1	.961		
Likelihood Ratio	.030	1	.862		
Fisher's Exact Test				.901	.479
Linear-by-Linear Association	.030	1	.862		
N of Valid Cases	1291				

a. 0 cells (0.0%) have expected count less than 5. The minimum expected count is 30.30.

b. Computed only for a 2x2 table

SBPCAT * AGECAT

Chi-Square Tests

	Value	df	Asymp. Sig. (2-sided)	Exact Sig. (2-sided)	Exact Sig. (1-sided)
Pearson Chi-Square	.298[a]	1	.585		
Continuity Correction[b]	.156	1	.693		
Likelihood Ratio	.297	1	.586		
Fisher's Exact Test				.651	.345
Linear-by-Linear Association	.298	1	.585		
N of Valid Cases	1287				

a. 0 cells (0.0%) have expected count less than 5. The minimum expected count is 20.19.

b. Computed only for a 2x2 table

DBPCAT * AGECAT

Chi-Square Tests

	Value	df	Asymp. Sig. (2-sided)	Exact Sig. (2-sided)	Exact Sig. (1-sided)
Pearson Chi-Square	1.025[a]	1	.311		
Continuity Correction[b]	.518	1	.472		
Likelihood Ratio	1.015	1	.314		
Fisher's Exact Test				.385	.235
Linear-by-Linear Association	1.024	1	.312		
N of Valid Cases	1287				

a. 0 cells (0.0%) have expected count less than 5. The minimum expected count is 5.27.

b. Computed only for a 2x2 table

QUIKICAT * AGECAT

Chi-Square Tests

	Value	df	Asymp. Sig. (2-sided)	Exact Sig. (2-sided)	Exact Sig. (1-sided)
Pearson Chi-Square	.127[a]	1	.722		
Continuity Correction[b]	.078	1	.780		
Likelihood Ratio	.127	1	.721		
Fisher's Exact Test				.756	.391
Linear-by-Linear Association	.127	1	.722		
N of Valid Cases	1313				

a. 0 cells (0.0%) have expected count less than 5. The minimum expected count is 87.29.

b. Computed only for a 2x2 table
