# Supplementary material for: Prevalence of hyperinsulinemia and its association with measures of adiposity and body composition in 16-25-year-old adolescents and young adults in Mumbai
Source: BMC Endocr Disord. 2026 Mar 13;26:117. doi: 10.1186/s12902-026-02230-0 (PMC13097659; doi:10.1186/s12902-026-02230-0)
Supplement: Supplementary file 2 — Supplementary Material 2 [file 12902_2026_2230_MOESM2_ESM.pdf]

Supplementary File 2: Comparison of variables between participants having different body weight status

		N	Mean	Std. Deviation	Std. Error	95% Confidence Interval for Mean Bound	Bound	Minimum	Maximum
WHR	1.00	306	.7607	.04942	.00280	.7552	.7662	.58	.90
	2.00	538	.7759	.05646	.00245	.7711	.7808	.63	1.09
	3.00	200	.7928	.05753	.00407	.7847	.8008	.67	.94
	4.00	269	.8177	.06868	.00419	.8095	.8260	.65	.98
	Total	1313	.7835	.06112	.00169	.7801	.7868	.58	1.09
WHtR	1.00	306	.3857	.02625	.00149	.3827	.3886	.32	.44
	2.00	538	.4294	.03251	.00141	.4267	.4322	.36	.56
	3.00	200	.4702	.02802	.00198	.4663	.4741	.40	.55
	4.00	269	.5348	.05597	.00341	.5281	.5416	.42	.79
	Total	1313	.4469	.06349	.00175	.4434	.4503	.32	.79
Mean SBP	1.00	306	101.27	10.199	.579	100.13	102.41	80	130
	2.00	538	104.24	10.782	.467	103.32	105.16	80	130
	3.00	200	106.83	11.333	.801	105.25	108.41	80	130
	4.00	269	109.84	12.221	.749	108.37	111.32	80	160
	Total	1313	105.07	11.423	.316	104.45	105.69	80	160
Mean DBP	1.00	306	64.62	7.624	.433	63.77	65.47	50	80
	2.00	538	66.08	7.741	.336	65.42	66.74	50	90
	3.00	200	67.65	7.923	.560	66.55	68.75	50	90
	4.00	269	69.79	8.591	.527	68.76	70.83	50	100
	Total	1313	66.73	8.116	.224	66.29	67.17	50	100
mean pulse	1.00	306	82.90	7.304	.415	82.08	83.71	60	103
	2.00	538	82.25	7.376	.320	81.62	82.88	60	111
	3.00	200	82.31	8.065	.572	81.18	83.44	59	119
	4.00	269	82.24	6.956	.425	81.41	83.08	50	110
	Total	1313	82.41	7.381	.204	82.01	82.81	50	119
Mean Body Fat	1.00	306	18.390	6.3397	.3595	17.683	19.097	5.0	33.8
	2.00	538	24.440	6.5634	.2843	23.881	24.998	5.0	34.0
	3.00	200	29.251	6.1260	.4332	28.397	30.105	17.1	37.1
	4.00	269	35.541	7.5235	.4587	34.638	36.444	14.3	51.3
	Total	1313	26.014	8.9000	.2456	25.532	26.496	5.0	51.3
Mean VF	1.00	306	1.055	.5662	.0347	.986	1.123	1.0	7.5
	2.00	538	2.903	1.4480	.0672	2.771	3.035	1.0	8.0
	3.00	200	5.577	1.5371	.1143	5.352	5.803	1.3	11.0
	4.00	269	8.460	2.5253	.1569	8.151	8.769	1.0	17.0
	Total	1313	4.125	3.1460	.0919	3.945	4.306	1.0	17.0

Mean muscle mass	1.00	306	33.459	6.1259	.3749	32.721	34.198	21.1	56.3
	2.00	538	37.470	6.9968	.3238	36.834	38.106	22.6	63.8
	3.00	200	41.209	8.0183	.5960	40.033	42.385	24.4	61.0
	4.00	269	44.153	9.4624	.5868	42.998	45.309	21.7	70.7
	Total	1313	38.613	8.4818	.2474	38.128	39.099	21.1	70.7
Sugr F	1.00	306	81.0984	8.32235	.47192	80.1698	82.0270	64.10	111.00
	2.00	538	80.8342	7.90580	.34341	80.1595	81.5088	62.20	114.00
	3.00	200	83.2470	10.65574	.75347	81.7612	84.7328	60.00	163.00
	4.00	269	84.5086	15.66316	.95678	82.6248	86.3924	61.20	287.90
	Total	1313	82.0179	10.55076	.29162	81.4458	82.5900	60.00	287.90
2 hr	1.00	306	88.0445	17.69350	1.00818	86.0607	90.0283	52.00	157.00
	2.00	538	91.5291	22.89226	.99532	89.5738	93.4844	44.00	376.00
	3.00	200	96.0365	25.52786	1.80509	92.4769	99.5961	50.00	304.20
	4.00	269	100.8474	33.26957	2.03226	96.8461	104.8487	54.60	513.00
	Total	1313	93.3111	25.15223	.69626	91.9452	94.6770	44.00	513.00
Ins F	1.00	306	6.73	4.827	.275	6.19	7.27	2	72
	2.00	538	7.89	4.105	.179	7.54	8.24	2	53
	3.00	200	9.93	6.686	.474	8.99	10.86	2	80
	4.00	269	11.70	6.264	.385	10.95	12.46	2	50
	Total	1313	8.70	5.513	.153	8.40	9.00	2	80
Ins 2hr	1.00	306	59.5820	47.60205	2.72123	54.2273	64.9368	2.80	412.00
	2.00	538	69.2971	54.22781	2.36444	64.6521	73.9420	0.00	396.00
	3.00	200	77.1447	57.09456	4.04733	69.1633	85.1261	5.00	350.00
	4.00	269	90.6180	68.01748	4.17042	82.4067	98.8294	2.90	400.00
	Total	1313	72.5818	57.29533	1.59092	69.4607	75.7028	0.00	412.00
HbA1C	1.00	118	5.423	.3236	.0291	5.366	5.481	4.6	6.2
	2.00	275	5.387	.3032	.0183	5.351	5.423	4.3	6.3
	3.00	110	5.400	.3604	.0344	5.332	5.468	4.6	8.0
	4.00	164	5.476	.5076	.0396	5.397	5.554	4.5	10.0
	Total	667	5.417	.3762	.0145	5.389	5.446	4.3	10.0
HOMAIRNEW	1.00	306	1.3383	.92098	.05222	1.2356	1.4411	0.00	12.55
	2.00	538	1.5682	.93275	.04040	1.4889	1.6476	0.00	11.99
	3.00	200	2.0688	1.69580	.11991	1.8323	2.3052	0.00	21.20
	4.00	269	2.4255	1.48096	.09030	2.2477	2.6032	0.00	10.75
	Total	1313	1.7656	1.26482	.03491	1.6972	1.8341	0.00	21.20
FGFIRATIO	1.00	306	14.7205	6.61201	.37493	13.9828	15.4583	0.00	42.80
	2.00	538	12.4005	6.38028	.27636	11.8576	12.9433	0.00	45.75
	3.00	200	10.7572	6.04934	.42775	9.9137	11.6007	0.00	42.45
	4.00	269	8.9901	5.44062	.33172	8.3370	9.6432	0.00	46.67
	Total	1313	12.0010	6.50738	.17959	11.6487	12.3533	0.00	46.67

QUIKI	1.00	306	.371350	.0466013	.0026425	.366151	.376550	0.0000	.4588
	2.00	538	.359511	.0527419	.0022845	.355023	.363999	0.0000	.4610
	3.00	200	.350922	.0388369	.0027462	.345507	.356337	0.0000	.4484
	4.00	269	.336132	.0539224	.0032877	.329659	.342605	0.0000	.4641
	Total	1313	.356217	.0510829	.0014098	.353452	.358983	0.0000	.4641

ANOVA

		Sum of Squares	df	Mean Square	F	Sig.
WHR	Between	.525	3	.175	52.328	.000
	Within	4.376	1309	.003		
	Total	4.900	1312			
WHtR	Between	3.518	3	1.173	866.378	0.000
	Within	1.772	1309	.001		
	Total	5.289	1312			
Mean SBP	Between	11526.481	3	3842.160	31.510	.000
	Within	159003.048	1304	121.935		
	Total	170529.529	1307			
Mean DBP	Between	4267.539	3	1422.513	22.669	.000
	Within	81826.654	1304	62.751		
	Total	86094.193	1307			
mean pulse	Between	96.125	3	32.042	.588	.623
	Within	71167.578	1305	54.535		
	Total	71263.703	1308			
Mean Body Fat	Between	45909.347	3	15303.116	345.290	.000
	Within	58014.322	1309	44.320		
	Total	103923.669	1312			
Mean VF	Between	8451.612	3	2817.204	1050.890	0.000
	Within	3128.470	1167	2.681		
	Total	11580.082	1170			
Mean muscle mass	Between	16901.083	3	5633.694	97.650	.000
	Within	67558.225	1171	57.693		
	Total	84459.309	1174			
Sugr F	Between	2970.292	3	990.097	9.059	.000
	Within	142634.230	1305	109.298		
	Total	145604.522	1308			
2 hr	Between	26929.705	3	8976.568	14.634	.000
	Within	798026.084	1301	613.394		
	Total	824955.789	1304			
Ins F	Between	4239.619	3	1413.206	51.972	.000
	Within	35240.351	1296	27.192		
	Total	39479.970	1299			

Ins 2hr	Between	148062.111	3	49354.037	15.540	.000
	Within	4106389	1293	3175.861		
	Total	4254451	1296			
HbA1C	Between	.849	3	.283	2.009	.111
	Within	94.237	669	.141		
	Total	95.087	672			
HOMAIRNEW	Between	213.051	3	71.017	49.294	.000
	Within	1885.854	1309	1.441		
	Total	2098.904	1312			
FGFIRATIO	Between	5133.255	3	1711.085	44.419	.000
	Within	50424.622	1309	38.521		
	Total	55557.877	1312			
QUIKI	Between	.191	3	.064	25.799	.000
	Within	3.232	1309	.002		
	Total	3.424	1312			

Post Hoc Tests

Multiple Comparisons

Bonferroni

Dependent Variable			Mean Difference (I-J)	Std. Error	Sig.	95% Confidence Interval	
						Lower Bound	Upper Bound
WHR	1.00	2.00	-.01527*	.00413	.001	-.0262	-.0044
		3.00	-.03207*	.00524	.000	-.0459	-.0182
		4.00	-.05706*	.00481	.000	-.0698	-.0443
	2.00	1.00	.01527*	.00413	.001	.0044	.0262
		3.00	-.01680*	.00479	.003	-.0295	-.0041
		4.00	-.04178*	.00432	.000	-.0532	-.0304
	3.00	1.00	.03207*	.00524	.000	.0182	.0459
		2.00	.01680*	.00479	.003	.0041	.0295
		4.00	-.02498*	.00540	.000	-.0392	-.0107
	4.00	1.00	.05706*	.00481	.000	.0443	.0698
		2.00	.04178*	.00432	.000	.0304	.0532
		3.00	.02498*	.00540	.000	.0107	.0392
WHtR	1.00	2.00	-.04376*	.00263	.000	-.0507	-.0368
		3.00	-.08454*	.00333	.000	-.0934	-.0757
		4.00	-.14917*	.00306	.000	-.1573	-.1411
	2.00	1.00	.04376*	.00263	.000	.0368	.0507
		3.00	-.04078*	.00305	.000	-.0488	-.0327
		4.00	-.10541*	.00275	.000	-.1127	-.0981
	3.00	1.00	.08454*	.00333	.000	.0757	.0934
		2.00	.04078*	.00305	.000	.0327	.0488
		4.00	-.06463*	.00343	.000	-.0737	-.0556

	4.00	1.00	.14917*	.00306	.000	.1411	.1573
		2.00	.10541*	.00275	.000	.0981	.1127
		3.00	.06463*	.00343	.000	.0556	.0737
Mean SBP	1.00	2.00	-2.971*	.789	.001	-5.06	-.89
		3.00	-5.562*	1.002	.000	-8.21	-2.92
		4.00	-8.574*	.923	.000	-11.01	-6.14
	2.00	1.00	2.971*	.789	.001	.89	5.06
		3.00	-2.591*	.916	.028	-5.01	-.17
		4.00	-5.603*	.829	.000	-7.79	-3.41
	3.00	1.00	5.562*	1.002	.000	2.92	8.21
		2.00	2.591*	.916	.028	.17	5.01
		4.00	-3.012*	1.033	.022	-5.74	-.28
	4.00	1.00	8.574*	.923	.000	6.14	11.01
		2.00	5.603*	.829	.000	3.41	7.79
		3.00	3.012*	1.033	.022	.28	5.74
Mean DBP	1.00	2.00	-1.456	.566	.061	-2.95	.04
		3.00	-3.027*	.718	.000	-4.93	-1.13
		4.00	-5.171*	.662	.000	-6.92	-3.42
	2.00	1.00	1.456	.566	.061	-.04	2.95
		3.00	-1.571	.657	.102	-3.31	.17
		4.00	-3.714*	.595	.000	-5.29	-2.14
	3.00	1.00	3.027*	.718	.000	1.13	4.93
		2.00	1.571	.657	.102	-.17	3.31
		4.00	-2.143*	.741	.023	-4.10	-.18
	4.00	1.00	5.171*	.662	.000	3.42	6.92
		2.00	3.714*	.595	.000	2.14	5.29
		3.00	2.143*	.741	.023	.18	4.10
mean pulse	1.00	2.00	.646	.528	1.000	-.75	2.04
		3.00	.586	.671	1.000	-1.19	2.36
		4.00	.651	.616	1.000	-.98	2.28
	2.00	1.00	-.646	.528	1.000	-2.04	.75
		3.00	-.060	.614	1.000	-1.68	1.56
		4.00	.005	.553	1.000	-1.46	1.47
	3.00	1.00	-.586	.671	1.000	-2.36	1.19
		2.00	.060	.614	1.000	-1.56	1.68
		4.00	.065	.691	1.000	-1.76	1.89
	4.00	1.00	-.651	.616	1.000	-2.28	.98
		2.00	-.005	.553	1.000	-1.47	1.46
		3.00	-.065	.691	1.000	-1.89	1.76
Mean Body Fat	1.00	2.00	-6.0498*	.4750	.000	-7.305	-4.795
		3.00	-10.8611*	.6034	.000	-12.456	-9.267
		4.00	-17.1511*	.5543	.000	-18.616	-15.686
	2.00	1.00	6.0498*	.4750	.000	4.795	7.305

		3.00	-4.8113*	.5520	.000	-6.270	-3.353
		4.00	-11.1012*	.4979	.000	-12.417	-9.786
	3.00	1.00	10.8611*	.6034	.000	9.267	12.456
		2.00	4.8113*	.5520	.000	3.353	6.270
		4.00	-6.2899*	.6216	.000	-7.932	-4.648
	4.00	1.00	17.1511*	.5543	.000	15.686	18.616
		2.00	11.1012*	.4979	.000	9.786	12.417
		3.00	6.2899*	.6216	.000	4.648	7.932
Mean VF	1.00	2.00	-1.8482*	.1259	.000	-2.181	-1.516
		3.00	-4.5228*	.1578	.000	-4.940	-4.106
		4.00	-7.4055*	.1429	.000	-7.783	-7.028
	2.00	1.00	1.8482*	.1259	.000	1.516	2.181
		3.00	-2.6747*	.1434	.000	-3.054	-2.296
		4.00	-5.5574*	.1269	.000	-5.893	-5.222
	3.00	1.00	4.5228*	.1578	.000	4.106	4.940
		2.00	2.6747*	.1434	.000	2.296	3.054
		4.00	-2.8827*	.1586	.000	-3.302	-2.463
	4.00	1.00	7.4055*	.1429	.000	7.028	7.783
		2.00	5.5574*	.1269	.000	5.222	5.893
		3.00	2.8827*	.1586	.000	2.463	3.302
Mean muscle mass	1.00	2.00	-4.0109*	.5828	.000	-5.551	-2.471
		3.00	-7.7495*	.7313	.000	-9.682	-5.817
		4.00	-10.6937*	.6618	.000	-12.443	-8.945
	2.00	1.00	4.0109*	.5828	.000	2.471	5.551
		3.00	-3.7386*	.6650	.000	-5.496	-1.981
		4.00	-6.6828*	.5877	.000	-8.236	-5.130
	3.00	1.00	7.7495*	.7313	.000	5.817	9.682
		2.00	3.7386*	.6650	.000	1.981	5.496
		4.00	-2.9442*	.7353	.000	-4.887	-1.001
	4.00	1.00	10.6937*	.6618	.000	8.945	12.443
		2.00	6.6828*	.5877	.000	5.130	8.236
		3.00	2.9442*	.7353	.000	1.001	4.887
Sugr F	1.00	2.00	.26424	.74677	1.000	-1.7089	2.2374
		3.00	-2.14861	.94759	.141	-4.6524	.3552
		4.00	-3.41019*	.87136	.001	-5.7126	-1.1078
	2.00	1.00	-.26424	.74677	1.000	-2.2374	1.7089
		3.00	-2.41285*	.86759	.033	-4.7053	-.1204
		4.00	-3.67443*	.78361	.000	-5.7450	-1.6039
	3.00	1.00	2.14861	.94759	.141	-.3552	4.6524
		2.00	2.41285*	.86759	.033	.1204	4.7053
		4.00	-1.26158	.97689	1.000	-3.8428	1.3196
	4.00	1.00	3.41019*	.87136	.001	1.1078	5.7126
		2.00	3.67443*	.78361	.000	1.6039	5.7450

		3.00	1.26158	.97689	1.000	-1.3196	3.8428
2 hr	1.00	2.00	-3.48463	1.77513	.299	-8.1750	1.2058
		3.00	-7.99202*	2.24911	.002	-13.9348	-2.0492
		4.00	-12.80291*	2.06889	.000	-18.2695	-7.3363
	2.00	1.00	3.48463	1.77513	.299	-1.2058	8.1750
		3.00	-4.50739	2.05585	.171	-9.9396	.9248
		4.00	-9.31828*	1.85697	.000	-14.2249	-4.4116
	3.00	1.00	7.99202*	2.24911	.002	2.0492	13.9348
		2.00	4.50739	2.05585	.171	-.9248	9.9396
		4.00	-4.81089	2.31425	.227	-10.9258	1.3041
	4.00	1.00	12.80291*	2.06889	.000	7.3363	18.2695
		2.00	9.31828*	1.85697	.000	4.4116	14.2249
		3.00	4.81089	2.31425	.227	-1.3041	10.9258
Ins F	1.00	2.00	-1.163*	.374	.011	-2.15	-.18
		3.00	-3.203*	.474	.000	-4.46	-1.95
		4.00	-4.978*	.437	.000	-6.13	-3.82
	2.00	1.00	1.163*	.374	.011	.18	2.15
		3.00	-2.040*	.434	.000	-3.19	-.89
		4.00	-3.815*	.393	.000	-4.85	-2.78
	3.00	1.00	3.203*	.474	.000	1.95	4.46
		2.00	2.040*	.434	.000	.89	3.19
		4.00	-1.775*	.489	.002	-3.07	-.48
	4.00	1.00	4.978*	.437	.000	3.82	6.13
		2.00	3.815*	.393	.000	2.78	4.85
		3.00	1.775*	.489	.002	.48	3.07
Ins 2hr	1.00	2.00	-9.71505	4.05171	.100	-20.4210	.9909
		3.00	-17.56270*	5.13203	.004	-31.1232	-4.0022
		4.00	-31.03602*	4.72419	.000	-43.5189	-18.5532
	2.00	1.00	9.71505	4.05171	.100	-.9909	20.4210
		3.00	-7.84765	4.69008	.567	-20.2404	4.5451
		4.00	-21.32097*	4.23994	.000	-32.5243	-10.1177
	3.00	1.00	17.56270*	5.13203	.004	4.0022	31.1232
		2.00	7.84765	4.69008	.567	-4.5451	20.2404
		4.00	-13.47332	5.28190	.065	-27.4298	.4832
	4.00	1.00	31.03602*	4.72419	.000	18.5532	43.5189
		2.00	21.32097*	4.23994	.000	10.1177	32.5243
		3.00	13.47332	5.28190	.065	-.4832	27.4298
HbA1C	1.00	2.00	.0365	.0406	1.000	-.071	.144
		3.00	.0234	.0492	1.000	-.107	.153
		4.00	-.0522	.0447	1.000	-.170	.066
	2.00	1.00	-.0365	.0406	1.000	-.144	.071
		3.00	-.0131	.0423	1.000	-.125	.099
		4.00	-.0887	.0370	.101	-.187	.009

	3.00	1.00	-.0234	.0492	1.000	-.153	.107
		2.00	.0131	.0423	1.000	-.099	.125
		4.00	-.0756	.0463	.616	-.198	.047
	4.00	1.00	.0522	.0447	1.000	-.066	.170
		2.00	.0887	.0370	.101	-.009	.187
		3.00	.0756	.0463	.616	-.047	.198
HOMAIRNEW	1.00	2.00	-.22989*	.08565	.044	-.4562	-.0036
		3.00	-.73042*	.10879	.000	-1.0179	-.4430
		4.00	-1.08714*	.09994	.000	-1.3512	-.8231
	2.00	1.00	.22989*	.08565	.044	.0036	.4562
		3.00	-.50053*	.09953	.000	-.7635	-.2375
		4.00	-.85725*	.08977	.000	-1.0944	-.6200
	3.00	1.00	.73042*	.10879	.000	.4430	1.0179
		2.00	.50053*	.09953	.000	.2375	.7635
		4.00	-.35671*	.11207	.009	-.6528	-.0606
	4.00	1.00	1.08714*	.09994	.000	.8231	1.3512
		2.00	.85725*	.08977	.000	.6200	1.0944
		3.00	.35671*	.11207	.009	.0606	.6528
FGFIRATIO	1.00	2.00	2.32010*	.44287	.000	1.1499	3.4903
		3.00	3.96335*	.56256	.000	2.4769	5.4498
		4.00	5.73047*	.51678	.000	4.3650	7.0960
	2.00	1.00	-2.32010*	.44287	.000	-3.4903	-1.1499
		3.00	1.64325*	.51467	.009	.2834	3.0031
		4.00	3.41038*	.46419	.000	2.1838	4.6369
	3.00	1.00	-3.96335*	.56256	.000	-5.4498	-2.4769
		2.00	-1.64325*	.51467	.009	-3.0031	-.2834
		4.00	1.76713*	.57949	.014	.2360	3.2983
	4.00	1.00	-5.73047*	.51678	.000	-7.0960	-4.3650
		2.00	-3.41038*	.46419	.000	-4.6369	-2.1838
		3.00	-1.76713*	.57949	.014	-3.2983	-.2360
QUIKI	1.00	2.00	.0118393*	.0035459	.005	.002470	.021209
		3.00	.0204283*	.0045042	.000	.008527	.032329
		4.00	.0352178*	.0041377	.000	.024285	.046151
	2.00	1.00	-.0118393*	.0035459	.005	-.021209	-.002470
		3.00	.0085889	.0041207	.224	-.002299	.019477
		4.00	.0233785*	.0037166	.000	.013558	.033199
	3.00	1.00	-.0204283*	.0045042	.000	-.032329	-.008527
		2.00	-.0085889	.0041207	.224	-.019477	.002299
		4.00	.0147895*	.0046397	.009	.002530	.027049
	4.00	1.00	-.0352178*	.0041377	.000	-.046151	-.024285
		2.00	-.0233785*	.0037166	.000	-.033199	-.013558
		3.00	-.0147895*	.0046397	.009	-.027049	-.002530

*. The mean difference is significant at the 0.05 level.
