# Supplementary material for: Prevalence of hyperinsulinemia and its association with measures of adiposity and body composition in 16-25-year-old adolescents and young adults in Mumbai
Source: BMC Endocr Disord. 2026 Mar 13;26:117. doi: 10.1186/s12902-026-02230-0 (PMC13097659; doi:10.1186/s12902-026-02230-0)
Supplement: Supplementary file 3 — Supplementary Material 3 [file 12902_2026_2230_MOESM3_ESM.docx]

**Supplementary Table 3: Comparison of the prevalence of hyperinsulinemia, insulin resistance, obesity, and hypertension between girls and boys in the study**

| Parameters | Total  (n=1313) | Girls  (n=856) | Boys  (n=457) | p-value |
| --- | --- | --- | --- | --- |
| Fasting insulin  Normal (<15mIU/ml)  Elevated (≥ 15 mIU/ml) | 1195 (91.1)  118 (9.0) | 769 (89.8)  87 (10.2) | 426 (93.2)  31 (6.8) | 0.041^*^ |
| Stimulated insulin  Normal (<80mIU/ml)  Elevated (≥ 80 mIU/ml) | 913 (69.5)  400 (30.5) | 544 (63.6)  312 (36.4) | 369 (80.7)  88 (19.3) | <0.001^**^ |
| Fasting glucose  Normal (<100mg/dL)  Elevated (100- 125mg/dL) | 1285 (97.9)  28 (2.1) | 839 (98.0)  17 (2.0) | 446 (97.6)  11 (2.4) | 0.633 |
| 2 -hour post glucose  Normal (<140mg/dl)  Elevated (140- 199 mg/dL) | 1282 (97.6)  31 (2.4) | 829 (96.8)  27 (3.2) | 453 (99.1)  4 (0.9) | 0.009^*^ |
| HbA1C (≤5.7%) ^#^  HbA1c (5.7-6.4%)  HbA1c (≥6.5%) | 533 (40.6)  131 (9.9)  3 (0.3) | 415 (48.5)  89 (10.4)  2 (0.2) | 118 (25.8)  42 (9.2)  1 (0.2) | 0.489 |
| HOMA IR >2.5 | 227 (17.3) | 155 (18.1) | 72 (15.8) | 0.129 |
| FG: FI ratio < 4.5 | 69 (5.3) | 48 (5.6) | 21 (4.6) | 0.439 |
| SBP ≥ 130 mmHg  DBP ≥ 80 mmHg | 46 (3.5)  12 (0.9) | 12 (1.4)  5 (0.6) | 34 (7.4)  7 (1.5) | <0.001^**^ |
| BMI < 22.9 kg/m^2^  BMI 22.9- 24.9 kg/m^2^  BMI >24.9 kg/m^2^ | 844 (64.3)  200 (15.2)  269 (20.5) | 549 (64.1)  121 (14.1)  186 (21.7) | 295 (64.6)  79 (17.3)  83 (18.2) | 0.134 |
| Waist to height ratio > 0.5 | 280 (21.3) | 168 (19.6) | 112 (24.5) | 0.038^*^ |

^#^n=667; ^*^ p <0.05; ^**^ p <0.001

HbA1c, Glycosylated Hemoglobin; HOMA-IR, Homeostasis Assessment Model- Insulin Resistance

FG: FI, Fasting Glucose: Fasting Insulin

SBP, Systolic Blood Pressure, DBP, Diastolic Blood Pressure, BMI, Body Mass Index

BMI 22.9- 24.9 kg/m^2^ is indicative of overweight, and BMI > 24.9 kg/m^2^ of obesity (WHO Asian Criteria)

Waist to height ratio >0.5 indicates central obesity
